# Artificial intelligence algorithm to predict the requirement of neonatal endotracheal intubation within 3 h: application for clinical practice

**DOI:** 10.3389/fmed.2026.1729990

**Published:** 2026-02-20

**Authors:** JinCheol Park, Minuk Yang, Ka Hyun Kim, Geun-Hyeong Kim, Seung Park

**Affiliations:** 1 College of Medicine, Chungbuk National University, Cheongju-si, Chungcheongbuk-do, Republic of Korea; 2 Development, Mediv Corp., Cheongju-si, Chungcheongbuk-do, Republic of Korea; 3 Department of Biomedical Engineering, Chungbuk National University Hospital, Cheongju-si, Chungcheongbuk-do, Republic of Korea

**Keywords:** deep learning, endotracheal intubation, multimodal network, neonatal intensive care units, neonates

## Abstract

**Introduction:**

Timely intervention, such as endotracheal intubation (EI), is crucial for managing acute respiratory distress in the neonatal intensive care unit (NICU). Delays in EI can lead to significant adverse effects in neonates. This study aimed to develop a highly accurate predictive model to forecast the requirement for EI, allowing for proactive clinical planning and intervention up to 3 h in advance.

**Method:**

We developed a multimodal deep learning model designed to simultaneously analyze distinct data types. The model utilizes numeric initial clinical data and time-series vital sign data collected over the preceding 1–3 h. To rigorously evaluate the model's reliability and clinical applicability, we performed comprehensive external validation using independent patient datasets, specifically assessing generalization and bias.

**Result:**

The constrained model successfully predicted the requirement for EI with high predictive power across various forecasting intervals (up to 72 h in 1-h increments). Internal validation yielded an accuracy of 0.9579 and AUC of 0.9323, while external validation maintained high generalization (accuracy 0.9411, AUC 0.9336).

**Discussion:**

The proposed multimodal deep learning model provides an effective tool for the advance prediction of EI requirements in neonates. Given its high accuracy, confirmed generalization capabilities through external validation, and potential to prevent severe respiratory distress problems by facilitating proactive care, this model holds wide and significant applicability in clinical NICU environments.

## 1 Introduction

Endotracheal intubation (EI) is a critical life-sustaining procedure for patients experiencing respiratory distress. Traditionally, EI decisions are made based on clinical assessment and available physiologic indicators, including vital signs and clinical scoring systems (1–3). Timely and accurate identification of newborns who are likely to require EI remains challenging in clinical practice because their clinical presentations and physiologic conditions can vary widely. In addition, delayed or inappropriate airway management in newborns with worsening respiratory failure can be associated with serious complications, including hypoxemia and bradycardia (4–6). Therefore, given these concerns, objective and quantifiable decision-support tools are needed to facilitate EI decision-making.

Recent studies have demonstrated that artificial intelligence (AI) models can support physicians in decision-making by predicting the optimal timing for EI based on patient data (7–10). To predict the need for EI at the time of patient admission, Nopour et al. (7) and Yu et al. (8) developed models based on multilayer perceptron (MLP) and eXtreme Gradient Boosting (XGBoost), respectively. Arvind et al. (9) utilized a random forest algorithm to predict EI requirements by analyzing time-series data, including blood pressure, respiratory rate, and oxygen saturation, at 12-h intervals over 3 days. Siu et al. (10) not only predicted the necessity of EI within 24 h for patients with respiratory distress but also identified key contributing variables in the model's decision-making process using Shapley additive explanations. Furthermore, Jueng-Eun et al. (11, 12) proposed AI models that can predict the timing of EI in 1-h intervals up to 12 h in advance. Their models leveraged initial clinical data from neonatal intensive care units (NICUs) along with hourly vital sign measurements for neonates experiencing respiratory distress.

However, previous studies (7–12) have three limitations. First, some studies (7, 8) do not reflect the health status of the patient over time. Physicians typically assess the condition of a patient by monitoring variations in vital signs, such as heart rate and oxygen saturation; however, these studies relied solely on initial clinical data obtained at the time of admission, without incorporating temporal changes in patient health. Second, the prediction time intervals set by previous studies (9–12) are limited. As delayed EI causes adverse effects, the prediction interval for EI should be short with the capability of long-term predictions. Nonetheless, previous studies (9, 10) either predicted EI application in 12-h intervals or began the prediction only 12 h after NICU admission. Moreover, although NICU patients are often hospitalized for extended periods, some studies (11, 12) only predicted EI application within the first 12 h after admission. Finally, existing models (7–10) may not be applicable to neonatal patients. Neonates exhibit distinct physiological characteristics in their respiratory and circulatory systems compared to adults (13, 14). As a result, EI prediction models originally designed for adult patients may not be suitable for neonatal use. Therefore, AI models should be developed to enable real-time assessment of patient health status, incorporate clinically relevant prediction intervals, and account for the unique physiological characteristics of neonates.

**Figure 1:**
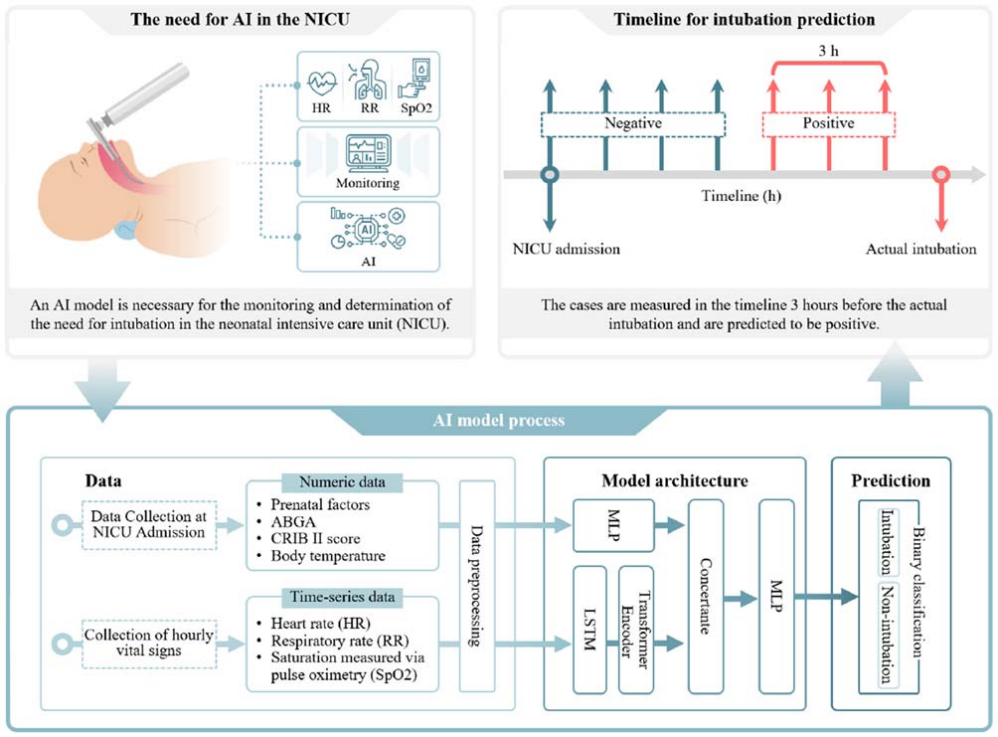
Overview of the development and validation process for an artificial intelligence model predicting the timing of endotracheal intubation (EI) in neonates.

To address these limitations, we developed a multimodal model that integrates two types of data: initial clinical data (numerical data) and vital signs (time-series data). An overview of the proposed workflow is shown in Figure 1. The model utilizes a multilayer perceptron (MLP) to process numerical data and a long short-term memory (LSTM)–transformer architecture to analyze time-series data. LSTM–Transformer is designed to capture micro-dynamics and inter-signal dependencies. To evaluate the model's performance and generalizability, we trained and internally validated it using a dataset of 432 patients from our institution, Chungbuk National University Hospital (CBNUH). External validation was conducted using a separate dataset of 176 patients from Chungnam National University Hospital (CNUH). Additionally, we extended the prediction window to 72 h after admission and enabled EI prediction at hourly intervals up to 3 h in advance, providing sufficient time for clinical preparation.

In summary, the contributions of our research are as follows:

- We developed an EI prediction model designed for neonatal patients.
- We validated the generalization of the proposed model using a dataset from an external institution.
- We predicted patients requiring EI at 1-h intervals and within 3 h, taking into account real-world clinical settings. Moreover, we extended the scope to predict neonatal patients up to 72 h after admission.

## 2 Method

### 2.1 Datasets

We collected the dataset for model training to predict EI and for internal validation at CBNUH, located in Cheong-Ju, Republic of Korea, between July 3, 2020, and August 4, 2023. The institutional review board (IRB) of CBNUH approved this study (IRB No. CBNUH 2021-02-034-001). For external validation, an additional dataset was collected at CNUH, located in Daejeon, Republic of Korea, between October 1, 2019, and June 30, 2021. This study was approved by the IRB of CNUH (IRB No. CNUH 2023-07-031). Both datasets were retrospectively collected through manual transcription of electronic medical records (EMRs), and all patient data were subsequently anonymized. We confirm that all methods were conducted in accordance with relevant ethical guidelines and regulations.

**Figure 2:**
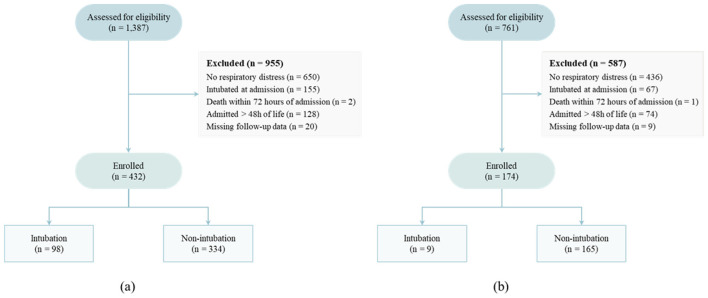
Flowchart of the data selection processes: **(a)** the dataset collected from Chungbuk National University Hospital (CBNUH) for development and internal validation; and **(b)** the dataset collected from Chungnam National University Hospital (CNUH) for external validation.

We established exclusion criteria to ensure the selection of a cohort relevant to the study objectives and maintain the integrity and reliability of the study results. The exclusion criteria included the following: (a) patients without dyspnea; (b) patients who had already underwent EI on admission to the NICU; (c) patients who had died within 72 h of admission; (d) patients hospitalized for more than 48 h after birth; and (e) patients with two or more missing numeric data. We selected the aforementioned criteria for the following reasons. Criteria (a)–(c) were not relevant to our study, which aimed to predict EI within 72 h of admission. Additionally, patients hospitalized more than 48 after birth were excluded due to an insufficient number of available data. Furthermore, since missing data exceeding 10% may cause bias in estimates (15, 16), we excluded patients with more than two missing numeric data. A total of 1,387 data points were collected from CBNUH, of which 955 cases were excluded based on the exclusion criteria, leaving 432 cases for model training and internal validation (Figure 2a). For the external validation, 761 data were collected from CNUH, with 587 cases excluded, resulting in 174 cases used for external validation (Figure 2b).

The collected variables included perinatal factors, arterial blood gas analysis (ABGA), patient vital signs, the Clinical Risk Index for Babies (CRIB) II score (17), and EI application status (Table 1). Perinatal factors comprised 12 variables: place of birth, gestational age, birth weight, gender, 1-min Apgar score, 5-min Apgar score, delivery type, antenatal steroid administration, pregnancy-induced hypertension, gestational diabetes mellitus, premature rupture of membranes, and multiple gestation. To assess neonatal clinical risk, we calculated the CRIB II score and used ABGA results that included pH, pCO_2_, pO_2_, base excess, and lactate concentration. All blood gas measurements included in the dataset were arterial samples as documented in the EMR; capillary blood gas analyses were not included. Additionally, four vital signs were collected: body temperature, heart rate (HR), respiratory rate (RR), and saturation measured via pulse oximetry (SpO_2_). Body temperature was recorded at admission, while HR, RR, and SpO_2_ were measured at 1-h intervals for up to 72 h. Hourly HR, RR, and SpO_2_ values were primarily transferred automatically from bedside monitoring devices to the EMR. If an hourly value was missing, we used the nearest nurse-charted value within ± 30 min of the target time point when available.

**Table 1:** Descriptions of the collected clinical variables in the dataset.

**Variable**		**Value [unit]**
Prenatal factors	Place of birth	0: Outside hospital, 1: In hospital
	Gestational age	204–296 [day]
	Birth weight	870–5,200 [g]
	1 min Apgar score	1–10
	5 min Apgar score	3–10
	Delivery type	0: Natural birth, 1: C-section
	Antenatal steroid administration	0: No, 1: Yes
	Pregnancy hypertension	0: No, 1: Yes
	Gestational diabetes mellitus	0: No, 1: Yes
	Premature rupture of membranes	0: No, 1: Yes
	Multi-birth	0: No multiples, 1: First, 2: Second, 3: Third or more
ABGA	pH	7.01–7.571
	pCO_2_	24.3–88.6 [mmHg]
	pO_2_	18.8–138 [mmHg]
	Base Excess	–16.5–18.5 [Mmol/L]
	Lactate	0.8–13.7 [Mmol/L]
Vital sign	HR	28–272 [Beats/min]
	RR	10–145 [Breaths/min]
	SpO_2_	10–100 [%]
	Body temperature	34–38.2 [°C]
CRIB II score	CRIB II score	1–10
Intubation	EI	0: Non-intubation, 1: Intubation

During the study period, neonatal respiratory management at both centers followed routine standard-of-care practices guided by the attending neonatologist; for transparency, we explicitly list the key clinical indications used to determine EI in the manuscript. EI was determined by the attending neonatologist based on common clinical indications, including persistent or worsening respiratory failure despite non-invasive respiratory support, recurrent apnea requiring ventilatory support, progressive respiratory acidosis or hypercapnia on ABGA, and the need for stabilization or airway protection.

The final collected dataset comprised both numerical and time-series data. To facilitate EI prediction within a 3-h window, we applied pre-processing methods tailored to each data type. The pre-processing steps for the numerical and time series data are described below.

#### 2.1.1 Numeric data pre-processing

##### 2.1.1.1 Nominal data encoding

We converted text-based nominal data (place of birth, sex, delivery type, antenatal steroid administration, gestational hypertension, gestational diabetes mellitus, preterm rupture of membrane, and multi-birth) into numeric data suitable for model analysis. The encoding results are shown in Table 1.

##### 2.1.1.2 Handling missing values in numeric data

Numeric data with more than two missing values were excluded from the dataset, ensuring that all retained numerical data contained at most one missing value. For this missing value, we replaced the nominal data (encoded as numerical data) with the mode value, and discrete and continuous data (gestational age, birth weight, 1-min Apgar score, 5-min Apgar score, pH, pCO_2_, pO_2_, base excess, lactate, and CRIB II score) with the mean value.

#### 2.1.2 Time-series data pre-processing

##### 2.1.2.1 Outlier handling in data

In time-series data analysis, outliers can adversely impact model performance. Therefore, zero values in the HR and RR data were treated as missing (NaN) and subsequently processed alongside other missing values. Since data from neonatal patients who died during hospitalization had already been excluded during data collection, zero values in HR and RR were identified as outliers measured likely recorded while the patient was crying or sleeping.

##### 2.1.2.2 Handling missing values in time-series data

In the collected dataset, 46.9% (46/98) of positive cases occurred within the first 3 h of hospitalization. Therefore, we applied a different imputation method for missing values based on 3 h. For time-series data recorded within the first 3 h, we imputed missing values by backward filling. This approach was chosen because the short data intervals between 0 and 2 h made it challenging to apply interpolation-based methods. For data after 3 h, we applied linear interpolation, which is a conventional method for handling missing values.

##### 2.1.2.3 Feature labeling in data and extraction of the last 3 h

**Figure 3:**
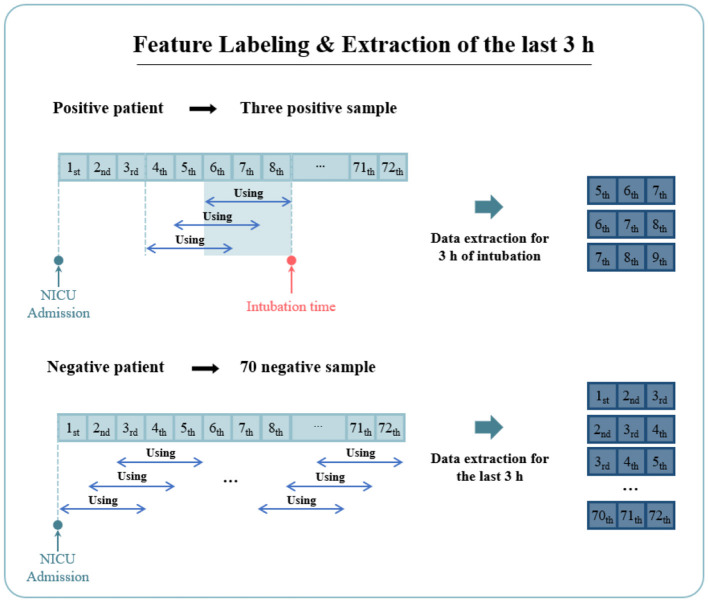
The process of pre-processing time-series data: Positive data were labeled as positive for data point corresponding to 3 h before the intubation time, and negative data point were labeled as negative for data in 1-h intervals.

The collected dataset contained information on whether intubation was performed within 72 h after NICU admission. As shown in Figure 3, we labeled the data features to enable the model to predict the need for intubation within a 3-h window. For positive cases, data points within 3 h prior to intubation were labeled as intubation. Data points recorded more than 4 h before intubation were excluded from model training to improve prediction accuracy. This exclusion was necessary because such data points could introduce ambiguity, potentially indicating the possibility of EI even in cases labeled as negative. Conversely, for negative cases, all data points were labeled as non-intubation. Across all time intervals, we extracted the last 3 h of data for each data sample and used it to train the model.

#### 2.1.3 Z-score standardization

Finally, we applied z-score standardization to all numerical and time-series data. This method transforms the data into a standard normal distribution, where the mean is zero and the standard deviation is one, as follows:


$$\begin{array}{l} Z=\frac{x-\mu }{\sigma } \end{array}$$ (1)

where *x* represents an individual data point, μ denotes the mean, and σ represents the standard deviation of the corresponding data. However, for time-series data, the z-score was calculated using the mean and standard deviation of HR, RR, and SpO_2_ over the 0—72 h period for each patient individually. This approach was chosen because calculating the mean and standard deviation across the entire patient population would obscure individual temporal trends, making it difficult to capture patient-specific variations over time.

### 2.2 Model architecture

**Figure 4:**
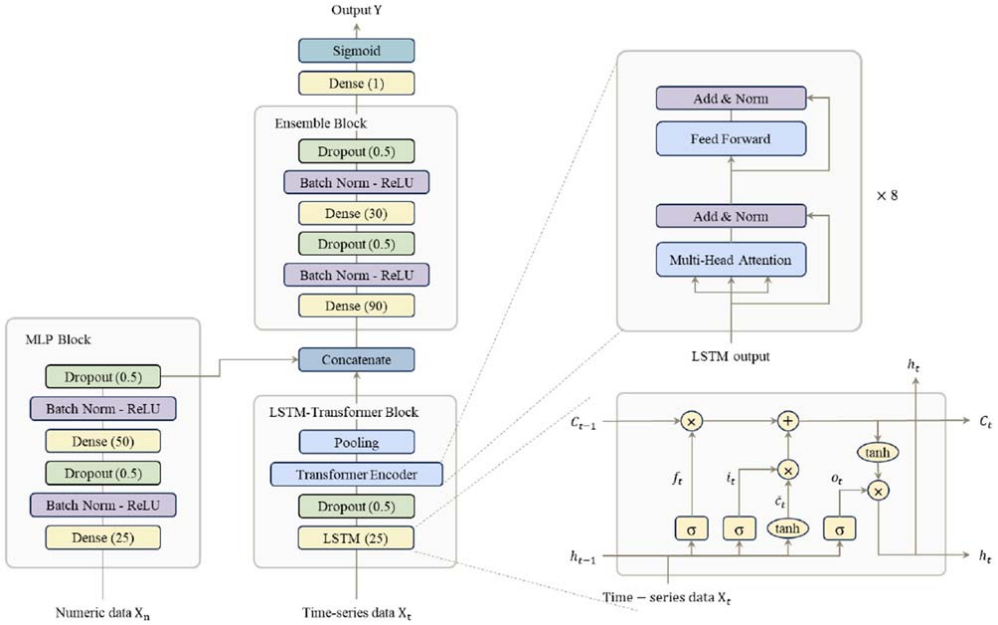
The model processes and integrates two types of data: numerical and time-series data. (1) Numerical data is processed through a Multi-Layer Perceptron (MLP) block to extract relevant features. (2) Time-series data is processed through a Long Short-Term Memory (LSTM)-Transformer block, where time-dependent features are extracted. The feature vectors from both branches are then passed through an Ensemble block, where they are concatenated to form the final model representation. This multimodal approach enables the model to effectively capture and integrate key information from both data types, enhancing overall predictive performance.

This section describes the multimodal model for predicting the probability of EI in NICU patients. As shown in Figure 4, to analyze the heterogeneous data, we designed a multimodal model for the integrated analysis of numeric and time-series data. Unlike previous MLP–LSTM (18, 19) or MLP–Transformer (20) models, our architecture explicitly combines both temporal dependency modeling (via LSTM) and inter-signal attention (via Transformer), improving robustness against irregular NICU measurements. The numeric and time-series data are analyzed using the MLP and LSTM-transformer blocks, respectively. As we expected the LSTM model to help in modeling the relationship between individual time-series data such as HR, RR, and SpO_2_, by considering their temporal dependence. Furthermore, transformer models help elucidate complex patterns by analyzing the interactions between data at each time step (21, 22). Therefore, this combination of LSTM and transformer models to aid in the comprehensive analysis of complex patterns in time-series data, including various features such as HR, RR, and SpO_2_. Moreover, as the LSTM replaces the positional encoding of the transformer, the model did not compute all the time information; conversely, it only computed the new temporal information per time step, thus reducing the computation time (23). The feature vectors analyzed by the MLP and LSTM–transformer blocks are concatenated and the probability of EI requirement is calculated in the ensemble block.

#### 2.2.1 MLP block

The MLP block processes numerical data *X*_n_, including initial clinical variables. It consists of two dense layers with 25 and 50 units, respectively, rectified linear unit (ReLU) activation, and dropout layers, which output feature vectors.

#### 2.2.2 LSTM-transformer block

To analyze time-series data, this study employed a multimodal LSTM-Transformer model (21, 22), which integrates Long Short-Term Memory (LSTM) (24) and transformer (25) architectures. Although each input sequence covers only a 3-h window, the model was designed to capture micro-dynamics and multi-signal dependencies (e.g., HR–SpO_2_ coupling) that simple LSTM networks may fail to express effectively. This design enables the model to attend to subtle temporal interactions and inter-feature correlations that can precede rapid physiological deterioration. The LSTM-Transformer block processes time-series data, denoted as *X*_t_ = (*X*_1_, *X*_2_, *X*_3_), which includes vital signs represented as three sequential features. The LSTM captures short-term temporal dependencies, while the Transformer attends to inter-signal correlations among HR, RR, and SpO_2_. The time-series data *X*_t_ includes a 3 h time window with three vital signs: heart rate (HR), respiratory rate (RR), and SpO_2_. These features are passed through the LSTM, generating a three-dimensional output with 25 hidden units, which is subsequently fed into the transformer component.

In this architecture, the LSTM replaces positional encoding in the transformer model (25). Since the transformer decoder is primarily designed for generative tasks such as machine translation, only the encoder is utilized in this model. The transformer encoder consists of two key components. First, Multi-head attention, which captures diverse dependencies within the input sequence. Second, a feedforward neural network (FFN), responsible for extracting features and recognizing patterns. The multi-head attention mechanism operates in parallel across eight attention heads, enabling the model to learn intricate relationships within the time-series data. The FFN includes layer normalization (computed using the mean and variance of input features), a dense layer with 50 units, GELU activation, and dropout, introducing non-linearity and improving the model's ability to learn complex representations. Finally, the sequence and embedding of time-series variables *X*_t_ processed by the transformer encoder are integrated into a unified output, effectively capturing both short-term and long-term dependencies in the time-series data.

#### 2.2.3 Ensemble block

The output feature vectors, having the same shape, from both the MLP and LSTM-Transformer blocks are concatenated to form a unified output vector, which is then used to compute the probability of requiring EI in the ensemble block. The ensemble block consists of two dense layers with 90 and 30 units, respectively, ReLU activation, and two dropout layers. Finally, the output. Finally, the output Y from the ensemble block is passed through a dense layer followed by a sigmoid activation function, generating the probability of requiring EI as a value ranging from 0 to 1.

### 2.3 Model training and implementation details

For model optimization, the Adam optimizer was employed with β_1_= 0.9 and β_2_= 0.999 and an exponential learning rate decay was applied over 100 steps. The initial learning rate and decay rate were set to 5 × 10^−5^ and 0.96, respectively. The model was trained for 2,000 epochs with a batch size of 1,024. During training, model checkpoints were saved based on the configuration that achieved the highest F1-score. To mitigate the effect of class imbalance, a weighted binary cross-entropy loss function was applied, assigning a weight of 3 to the intubated (EI) class and 1 to the non-EI class to penalize misclassification of the minority class. The proposed model was implemented using TensorFlow 2.4.1 with Python 3.8 and trained on an NVIDIA RTX 3090 GPU (CUDA v.11.0) with an Intel^®^ Core i7-12700 CPU.

### 2.4 Performance evaluation

We employed 4-fold cross-validation to improve the model's generalization ability and mitigate overfitting.To quantitatively assess the performance of the trained model in each fold, we evaluated accuracy, sensitivity, specificity, the receiver operating characteristic (ROC) curve, and the area under the ROC curve (AUROC). The ROC curve represents the relationship between 1-specificity (false positive rate) and sensitivity (true positive rate). The evaluation metrics were calculated using the following equation.


$$\begin{array}{l} \text{Accuracy}=\frac{\text{TP }+\text{ TN}}{\text{TP }+\text{ TN }+\text{ FP }+\text{ FN}} \end{array}$$ (2)

$$\begin{array}{l} \text{Sensitivity}=\frac{\text{TP}}{\text{TP }+\text{ FN}} \end{array}$$ (3)

$$\begin{array}{l} \text{Specificity}=\frac{\text{TN}}{\text{TN }+\text{ FP}} \end{array}$$ (4)

Each of the four trained models utilized the Youden index (J) to determine the optimal threshold for its respective fold, balancing both sensitivity and specificity. Sensitivity and specificity were computed for each threshold and plotted on the receiver operating characteristic (ROC) curve. The threshold that maximized the J value, derived from the sensitivity and specificity at each threshold, was selected as the optimal threshold. J is calculated as follows:


$$\begin{array}{l} \text{J}=\text{Sensitivity }+\text{ Specificity}-1 \end{array}$$ (5)

## 3 Result and discussion

**Figure 5:**
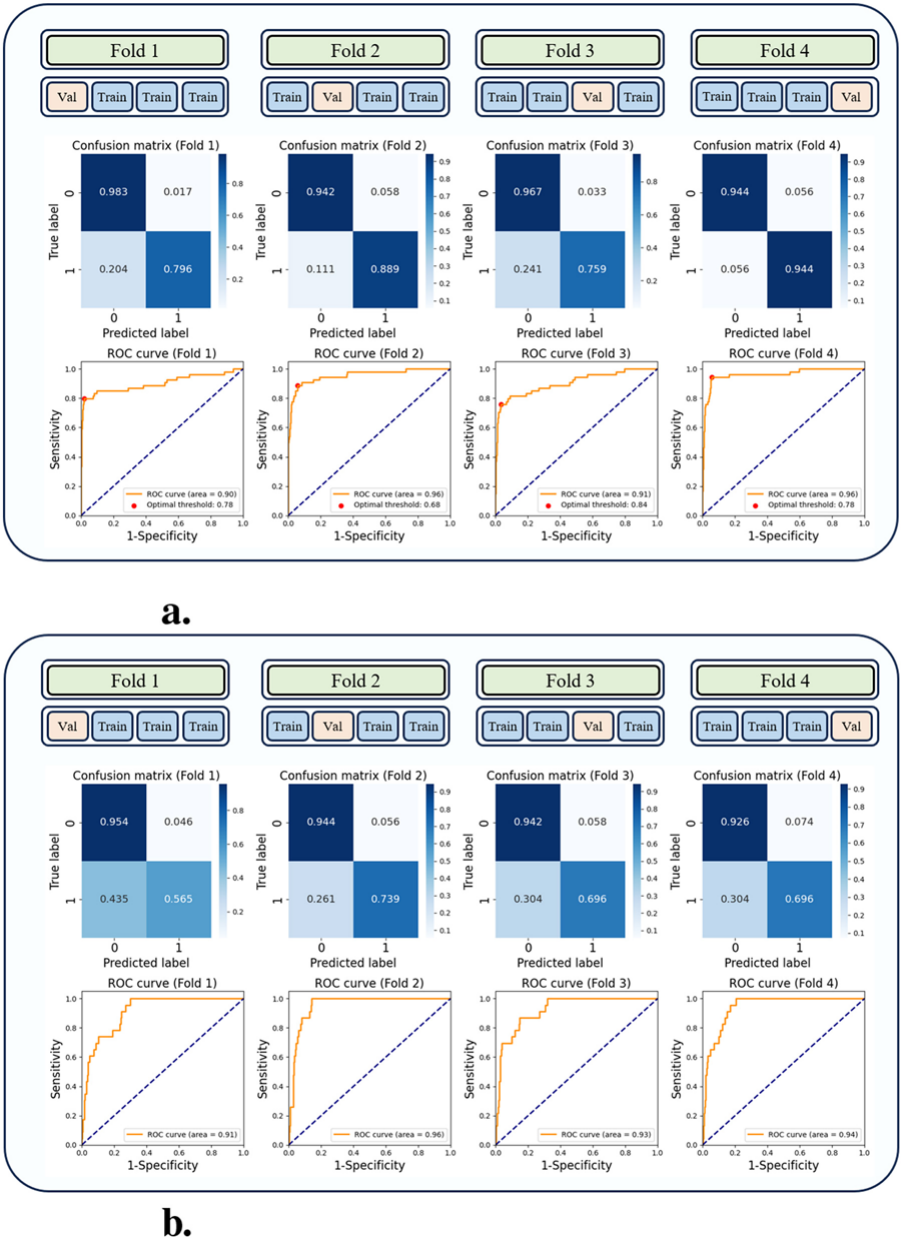
Confusion matrices and receiver operating characteristic (ROC) curves of the EI prediction model, calculated through four-fold cross-validation. **(a, b)** depict the results for each fold using the test sets from the interval and external datasets, respectively. **(a)** Internal validation. **(b)** External validation.

The proposed model was compared with other models used in recent EI applied prediction studies [random forest (9, 10), logistic regression (7, 10), and MLP (7)], other machine learning (ML) models (light gradient boosted machine (LightGBM)), and multimodal models similar to the proposed model [MLP-LSTM (18, 19) and MLP-transformer (12, 20)]. ML models (Random Forest, Logistic Regression, and LightGBM) were implemented using the scikit-learn library and optimized via grid search. The grid search included tuning hyperparameters such as the number of estimators for Random Forest, the regularization strength for Logistic Regression, and the learning rate and number of leaves for LightGBM. Deep learning models (MLP, MLP-LSTM, and MLP–transformer) were trained on numeric and time-series data and optimized via randomized search. The performances of each model are summarized in Table 2. As shown in Table 2 and Figure 5a, the proposed model achieved the following performances in the four averaged metrics: accuracy = 0.9579 ± 0.02, sensitivity = 0.8472 ± 0.08; specificity = 0.9591 ± 0.02; AUROC = 0.9323 ± 0.03. The proposed model showed the best performance in all evaluation metrics as compared to those of previous studies (7, 9, 10) and ML models. Furthermore, when comparing the evaluation metrics of the proposed model with other models based on multimodal architecture (12, 18–20), the proposed model demonstrates the best results in terms of accuracy, specificity, and AUROC, as well as the second-best performance in sensitivity. The MLP-LSTM model performed second best, with a difference of within 2% for each performance metric compared to the proposed model. Most models showed low performance in sensitivity and were weak at predicting positive cases.

**Table 2:** Performance of the proposed model, machine learning (ML) models, and deep learning (DL) models on internal and external validation datasets.

**Model**		**Accuracy**	**Sensitivity**	**Specificity**	**AUROC**
Internal validation	Random forest	0.9386 ± 0.02	0.6535 ± 0.04	0.9420 ± 0.02	0.9113 ± 0.01
	Logistic regression	0.8940 ± 0.01	0.6930 ± 0.10	0.8964 ± 0.01	0.8854 ± 0.01
	LightGBM	0.9091 ± 0.02	0.6711 ± 0.21	0.9119 ± 0.02	0.8881 ± 0.04
	MLP	0.8572 ± 0.07	0.8426 ± 0.10	0.8574 ± 0.07	0.9120 ± 0.04
	MLP-LSTM	0.9417 ± 0.04	**0.8565** **±** **0.08**	0.9427 ± 0.04	0.9282 ± 0.03
	MLP-transformer	0.9313 ± 0.03	0.8426 ± 0.08	0.9323 ± 0.03	0.9211 ± 0.02
	Proposed model	**0.9579** **±** **0.02**	0.8472 ± 0.08	**0.9591** **±** **0.02**	**0.9323** **±** **0.03**
External validation	Random forest	0.7472 ± 0.07	0.6196 ± 0.04	0.7474 ± 0.07	0.7483 ± 0.04
	Logistic regression	0.5160 ± 0.14	**0.8913** **±** **0.08**	0.5153 ± 0.14	0.8091 ± 0.00
	LightGBM	0.4314 ± 0.21	0.7283 ± 0.22	0.4308 ± 0.21	0.6295 ± 0.08
	MLP	0.8066 ± 0.09	0.6196 ± 0.03	0.8070 ± 0.09	0.7967 ± 0.05
	MLP-LSTM	0.9220 ± 0.06	0.5761 ± 0.26	0.9227 ± 0.06	0.8813 ± 0.03
	MLP-transformer	0.9283 ± 0.04	0.4674 ± 0.05	0.9292 ± 0.04	0.7784 ± 0.04
	Proposed model	**0.9411** **±** **0.01**	0.6739 ± 0.08	**0.9416** **±** **0.01**	**0.9336** **±** **0.02**

The best and second-best performances are denoted by bold and underlined, respectively.

**Figure 6:**
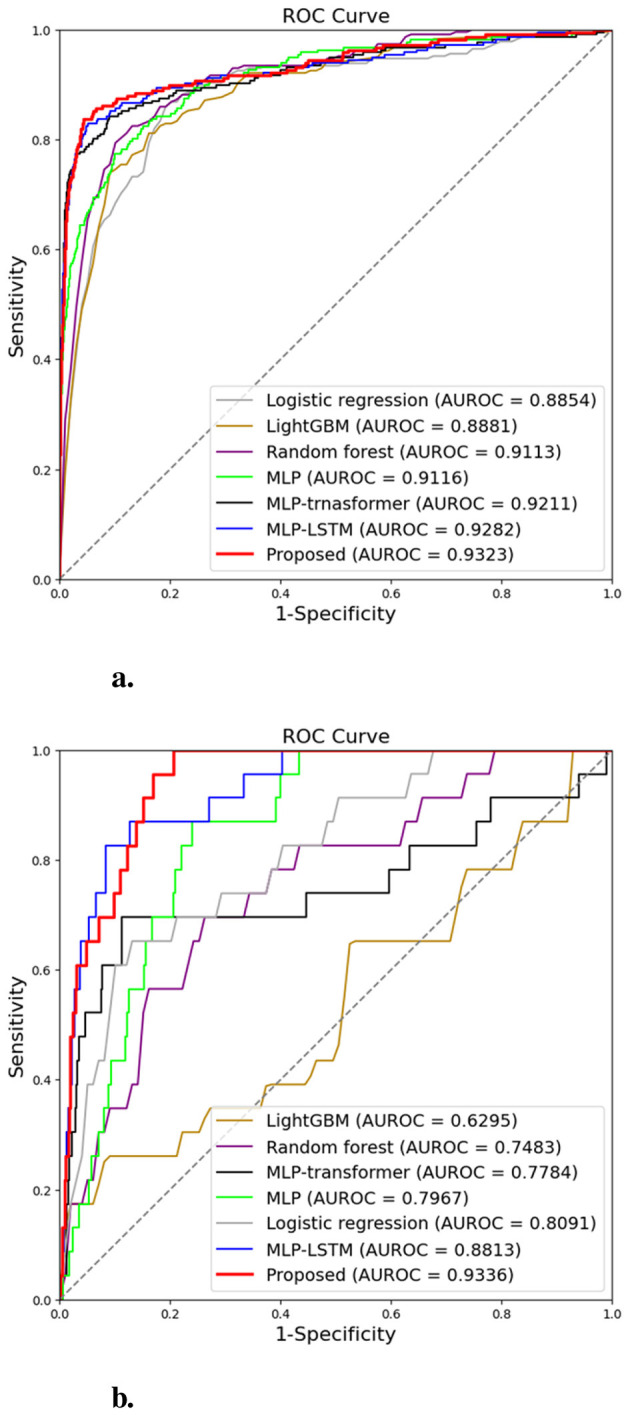
Average receiver operating characteristic (ROC) curves of the proposed and comparative models obtained through four-fold cross-validation. **(a)** ROC curves for the internal validation datasets. **(b)** ROC curves for the external validation datasets.

To validate the generalization of the proposed model, external validation was performed using datasets collected from other institutions. The results were summarized using a confusion matrix and ROC curve as shown in Figure 5b. We compared the external validation results of the proposed model with those of other models, and the results of the evaluation metrics are listed in Table 2, while comparative results of the ROC curve are shown in Figure 6b. As listed in Table 2, the proposed model achieved the following performances in the four average metrics on the external dataset: accuracy = 0.9411 ± 0.01; sensitivity = 0.6739 ± 0.08; specificity = 0.9416 ± 0.01; AUROC = 0.9336 ± 0.02. The proposed model demonstrated good performance in accuracy, specificity and AUROC when compared with previous studies (7, 9, 10) and ML models in external validation. Logistic regression and LightGBM showed sensitivity 0.8913 ± 0.08 and 0.7283 ± 0.22, respectively, in the external validation results. However, they exhibited a bias toward positive cases predictions attributed to their low accuracy and specificity. The proposed model achieved the third-highest sensitivity, and excluding the two models that exhibited a bias toward positive case predictions, it showed the highest sensitivity among the remaining models. Moreover, we compared the evaluation metrics of the proposed model with other multimodal-based models (12, 18–20) and found that it performs well on all evaluation metrics. Deep learning-based models (MLP, MLP-LSTM, MLP-transformer, and proposed model) demonstrated good performance in accuracy, specificity, and AUROC, but exhibited relatively low values in sensitivity.

**Figure 7:**
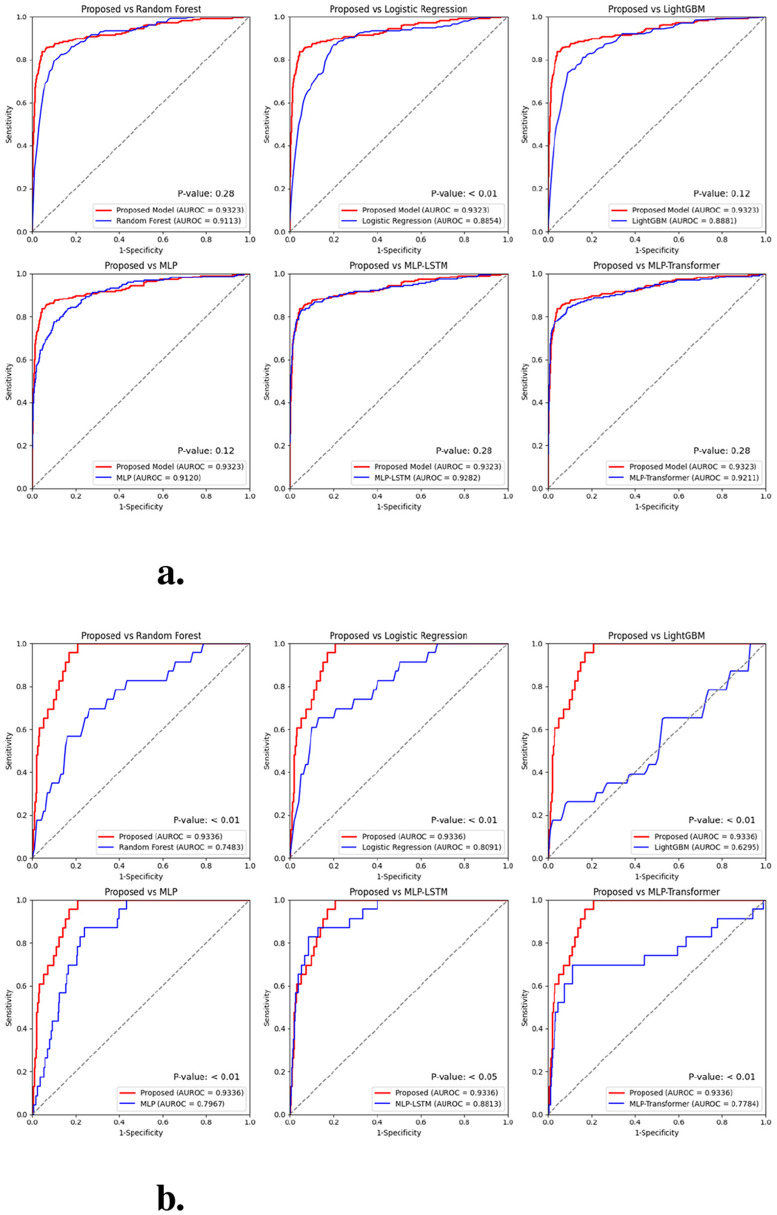
Results of the non-parametric permutation test comparing the ROC curves of the proposed model and the comparison models: **(a)** AUC in internal validation datasets; **(b)** AUC in external validation datasets.

Figure 6 shows the ROC curves comparing the proposed model with other baseline models. The proposed model showed superior AUC and ROC curve performance in both internal and external validation. Furthermore, in Figure 7, to compare the classification results between the proposed model and the baseline models, we performed a non-parametric permutation test of the ROC curves (26). In internal validation(in Figure 7a), all models achieved similar AUROC values (0.91—0.93) with no statistically significant difference (*p*-value>0.05). This suggests that most models could effectively learn the underlying patterns within the same institutional dataset. In contrast, in the external validation(in Figure 7b) using data from a different institution, the proposed model demonstrated a statistically significant improvement (*p*-value < 0.05) compared to other baselines. Despite differences in data distribution and measurement frequency, the model maintained high AUROC (0.93) and accuracy (0.94), indicating strong generalization performance under domain shift conditions. This robustness suggests the model's potential for multi-institutional deployment. This finding implies that the hybrid LSTM–Transformer architecture effectively captured robust temporal dependencies and cross-feature interactions, which conventional models such as random forest or MLP-based approaches failed to generalize.

However, as shown in Table 2 and Figure 6, the model performance was lower in the external validation (sensitivity = 0.8472) as compared to that in the internal validation (sensitivity = 0.6739). This was because of two issues with the dataset collected for external validation. First, the external institution that collected the dataset used for external validation had a high number of missing positive cases owing to the institutional tendency of not recording vital sign data on an immediately before applying EI to a patient. This resulted in a significantly unbalanced dataset between positive and negative data (positive cases: 14, negative cases: 165). This can be addressed by focusing on collecting more positive cases during future data collection. Second, the datasets we collected had many missing RRs: the training dataset was collected at hourly intervals, while the external institution dataset was collected at irregular time intervals. Moreover, both internal and external validation results indicate that sensitivity showed lower performance compared to accuracy, specificity, and AUROC. This is the result of a trade-off between sensitivity and specificity, which can be resolved by adjusting the numbers.

**Figure 8:**
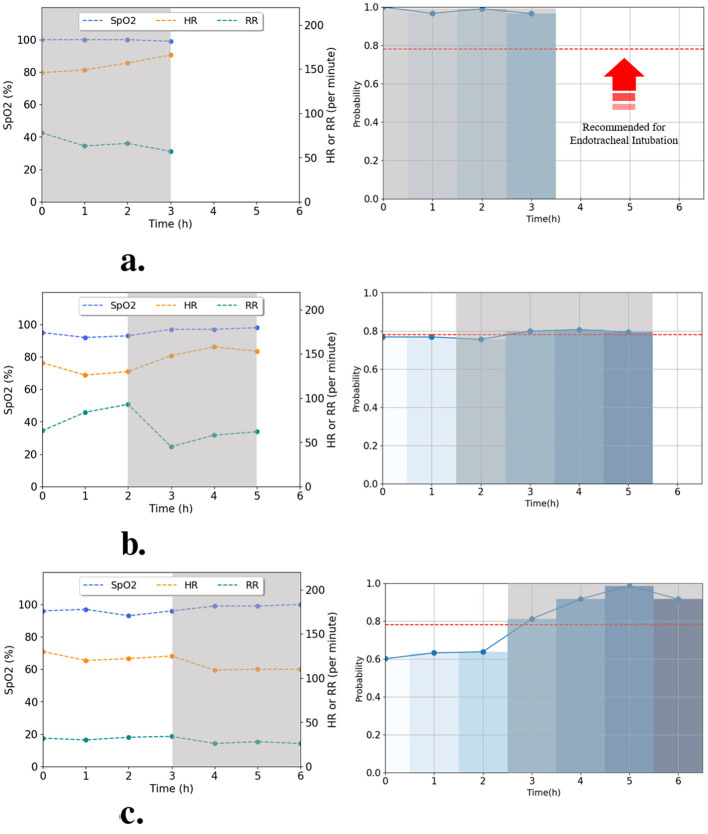
Real-time prediction for intubated patients using the best-performing fold 4 model. Graphs **(a–c)** illustrate the time-series data (left) and predicted intubation probability (right) for patients who were intubated after 3, 5, and 6 h, respectively. The predicted probabilities are classified according to the optimal threshold (horizontal dotted lines). **(a)** Patient case intubated after 3 h. **(b)** Patient case intubated after 5 h. **(c)** Patient case intubated after 6 h.

**Figure 9:**
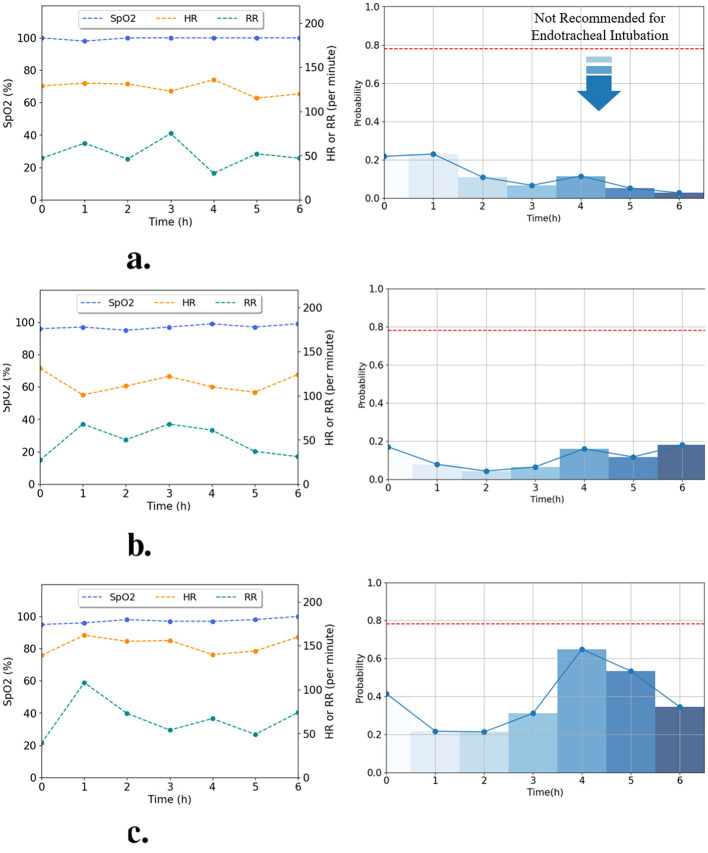
Real-time prediction for non-intubated patients using the best-performing fold 4 model. Graphs **(a–c)** illustrate the time-series data (left) and predicted probability (right) for patients without intubation. The model consistently predicted below the optimal threshold (horizontal dotted lines), confirming the non-intubation outcome. **(a)** Patient case without intubation 1. **(b)** Patient case without intubation 2. **(c)** Patient case without intubation 3.

In this study, the proposed model offers predictions of intubation probabilities using multimodal information including 3 h of vital signs and initial clinical variables. To develop and evaluate this model, we established a dataset including information on 432 neonatal patients and evaluated a multimodal model that could classify neonates requiring intubation 3 h in advance. The model used for validation was validated using fold 4, which showed the best performance metrics among the four trained folds. The AI model recommended EI if the probability exceeded 78.1% (optimal threshold of fold 4); otherwise, it did not recommend EI. In Figures 8, 9, the process of determining the probability of predicting EI, alongside the vital signs (HR, RR, and SpO_2_) over time, is depicted based on the data of six random patients using the proposed model is shown. Figure 8 illustrate the intubation cases where EI was applied either 3, 5, or 6 h after admission, respectively. Figure 9 show the non-intubation case. Figure 8a shows that from 0 to 3 h after admission, HR increased by approximately 7.64%, RR decreased by approximately 15.79%, and SpO_2_ showed minimal change in value. The probability of EI occurring during this period varied from 78% to 97%. As evident from Figure 8b, the RR decreased sharply to 51.6% between the 2 and 3 h time of admission, and the change gradually stabilized from 3 to 5 h; therefore, the probability of applying EI went down reduced by approximately 5.5%. Furthermore, during at 2–3 h after admission, the RRs was 80 and 74 breaths per min, respectively, which were out of the normal range (normal range of RR: 30–60 breaths per min). In Figure 8c, HR decreased by 12.8% between 4 and 6 h to 109–110 beats per min, which was above the normal range (normal range of HR: 120–160 beats per min). From 4–6 h, the HR remained low, and the probability of EI increased from 63.2% to 98.6%. Figure 9 show that the HR and RR values often appeared to deviate from the normal range. Furthermore, the vital signs changed rapidly and the probability of applying EI increased or decreased. However, we confirmed that the predictive probability of intubation was stable. This appears to reflect the analysis results of numeric data because our model integrates numeric and time-series data. Figures 8, 9 were designed not only to visualize prediction dynamics but also to provide case-based interpretability, showing that the predicted EI probability follows clinically plausible vital sign trajectories.

## 4 Limitations

The proposed model has three primary limitations in analyzing time-series data.

First, it exhibits bias when predicting short sequences. The model processes sequences up to 3 h in 1-h increments. Among the positive cases collected, 31.6% (31/98) underwent EI within 2 h of NICU admission. Consequently, 31.3% of patients had missing data due to backfilling, which may have introduced bias in the analysis. Therefore, further research is needed to develop a model specifically designed to analyze short-sequence vital signs data and integrate it into the existing framework. Accordingly, predictions generated immediately after NICU admission, when substantial backfilling may be required, should be interpreted with caution.

Second, the proposed model heavily relies on time-series data. The dataset was augmented in 1-h intervals, and the last 3 h of data were extracted. While the proposed model utilizes separate encoders for static and time-series inputs, the window-based augmentation replicates static variables across multiple time steps, which may increase the model's reliance on time-series patterns. Future work will explore fusion strategies (e.g., modality gating or modality-specific regularization) to better balance contributions from static and temporal features.

Third, to preserve the intrinsic temporal dependencies and physiological consistency of the time-series data, no synthetic oversampling techniques (e.g., SMOTE) were applied, even though there was a notable class imbalance between the intubated (positive) and non-intubated (negative) groups. Instead, the imbalance was indirectly compensated by applying a weighted binary cross-entropy loss during model training, which penalized misclassification of the minority (intubated) class more heavily. In addition, while we report performance using an optimal threshold determined by the Youden index, the operating threshold can be calibrated to prioritize sensitivity for early warning applications depending on institutional clinical priorities. Regarding external validation, the reduced sensitivity observed in external validation may reflect a limited number of positive cases and outcome-dependent missingness (e.g., incomplete near-event vital-sign capture) as well as irregular charting intervals (particularly for RR) at the external site. Future external validation should adopt prospective multi-center protocols that ensure comprehensive vital-sign recording immediately preceding EI and investigate approaches robust to irregular sampling (e.g., time-aware model inputs and enhanced interpolation methods).

## 5 Conclusion

We developed a real-time prediction system utilizing a multimodal model to predict neonatal EI 3 h in advance. The proposed model outperformed previously developed models, achieving the following results: accuracy = 0.9579 ± 0.02, sensitivity = 0.8472 ± 0.08, specificity = 0.9591 ± 0.01, and AUROC = 0.9323 ± 0.03. The proposed model also demonstrated superior performance in external validation using datasets collected from an independent institution, achieving: accuracy = 0.9411 ± 0.01, sensitivity = 0.6739 ± 0.08, specificity = 0.9416 ± 0.01, and AUROC = 0.9336 ± 0.02. Moreover, the model was designed to reflect real-world clinical applications by incorporating three prediction windows: hourly intervals, within 3 h, and up to 72 h after admission. We anticipate that the proposed model will contribute to the early prevention of respiratory distress by enabling timely EI predictions in neonates. Given its generalizability and effectiveness, the model holds significant potential for clinical implementation. In future clinical implementation, the model could be integrated into real-time NICU monitoring systems to provide early warnings to physicians when the predicted probability of EI exceeds the predefined threshold. This could allow pre-intubation preparation, reduce procedure-related complications, and improve neonatal outcomes.

## Data Availability

The datasets generated and/or analyzed during the current study are available from the corresponding author on reasonable request, subject to institutional approvals and data-sharing agreements.
